# Remdesivir–ivermectin combination displays synergistic interaction with improved *in vitro* activity against SARS-CoV-2

**DOI:** 10.1016/j.ijantimicag.2022.106542

**Published:** 2022-03

**Authors:** Laura N. Jeffreys, Shaun H. Pennington, Jack Duggan, Claire H. Caygill, Rose C. Lopeman, Alastair F. Breen, Jessica B. Jinks, Alison Ardrey, Samantha Donnellan, Edward I. Patterson, Grant L. Hughes, David W. Hong, Paul M. O'Neill, Ghaith Aljayyoussi, Andrew Owen, Stephen A. Ward, Giancarlo A. Biagini

**Affiliations:** a Centre for Drugs and Diagnostics, Department of Tropical Disease Biology, Liverpool School of Tropical Medicine, Liverpool, UK; b Department of Vector Biology, Liverpool School of Tropical Medicine, Liverpool, UK; c Department of Chemistry, University of Liverpool, Liverpool, UK; d Department of Pharmacology and Therapeutics, Centre of Excellence in Long-acting Therapeutics, University of Liverpool, Liverpool, UK

**Keywords:** SARS-CoV-2, COVID-19, Cytopathic activity, CPE, Combination therapy, Synergy

## Abstract

A key element for the prevention and management of coronavirus disease 2019 is the development of effective therapeutics. Drug combination strategies offer several advantages over monotherapies. They have the potential to achieve greater efficacy, to increase the therapeutic index of drugs and to reduce the emergence of drug resistance. We assessed the *in vitro* synergistic interaction between remdesivir and ivermectin, both approved by the US Food and Drug Administration, and demonstrated enhanced antiviral activity against severe acute respiratory syndrome coronavirus-2. Whilst the *in vitro* synergistic activity reported here does not support the clinical application of this combination treatment strategy due to insufficient exposure of ivermectin *in vivo*, the data do warrant further investigation. Efforts to define the mechanisms underpinning the observed synergistic action could lead to the development of novel treatment strategies.

## 1 Introduction

At the time of writing, the World Health Organization (WHO) has reported more than 328 million cases of coronavirus disease 2019 (COVID-19) and more than 5.5 million deaths [1]. There remains a clear need for therapeutic strategies with activity against severe acute respiratory syndrome coronavirus-2 (SARS-CoV-2). Potential therapeutic strategies may include the repurposing of existing drugs as well as the discovery of novel therapies. Thousands of clinical trials are currently underway, with therapeutic approaches involving direct-acting antivirals for the prevention of viral replication, and host-directed therapies aimed at mitigating against the disease pathology [2,3].

Combination therapies can offer several advantages over monotherapies. They have the potential to achieve greater efficacy, to increase the therapeutic index of drugs and to reduce the emergence of drug resistance. Strategies to identify effective combination therapies are emerging, with several laboratories reporting *in vitro* combination screens [4] and *in vivo* animal combination studies [5]. In a recent clinical trial, baricitinib administered in combination with remdesivir was found to be superior, and to elicit fewer adverse effects, compared with either drug in isolation [6]. Importantly, even in the absence of synergistic activity, an additive interaction between two drugs with separate mechanisms of action may profoundly reduce the speed at which drug resistance is established.

Both remdesivir and ivermectin have received attention for the treatment of COVID-19. Remdesivir is a prodrug C-adenosine nucleoside analogue that inhibits the viral RNA-dependent, RNA polymerase. Early in the pandemic, remdesivir was shown to display *in vitro* antiviral efficacy against SARS-CoV-2 [7]. In a double-blind, randomized, placebo-controlled trial, intravenous administration of remdesivir showed superiority relative to placebo in shortening the time to recovery in adults who were hospitalized with COVID-19 [8]. However, other studies indicated that its impact was negligible [9], and on 20 November 2020, WHO issued a conditional recommendation against the use of remdesivir in hospitalized patients (irrespective of disease severity) due to and absence of evidence supporting an improvement in survival or other outcomes in patients.

Ivermectin is an antiparasitic which is active against a wide range of parasites, including gastrointestinal roundworms, lungworms, mites, lice, hornflies and ticks [10]. Ivermectin is reported to exhibit broad-spectrum antiviral activity against a wide range of RNA and DNA viruses [11]. Recently, ivermectin was also shown to display antiviral activity against SARS-CoV-2 [12], but approved doses are not expected to be high enough to achieve *in vitro*-defined target exposures systemically [13]. Several clinical trials are now evaluating the potential of ivermectin for both prophylaxis and treatment of COVID-19, but low exposures make the anti-inflammatory and/or immunomodulatory mechanisms of action more plausible than direct antiviral activity of the monotherapy [14], particularly as studies with SARS-CoV-2 in Syrian golden hamsters showed an impact upon disease pathology in the absence of any effect on viral titres [15].

The authors found a synergistic interaction between remdesivir and ivermectin resulting in improved *in vitro* antiviral activity against SARS-CoV-2 using two distinct methodologies – determination of the fractional inhibitory concentration index (FICI) with isobologram analyses, and checkerboard combinations with SynergyFinder analyses. The data are discussed in the context of current therapeutic efforts against COVID-19.

## 2 Materials and methods

### 2.1 SARS-CoV-2 strain

SARS-CoV-2/Human/Liverpool/REMRQ0001/2020 was isolated from a nasopharyngeal swab from a patient in Liverpool and passaged a further four times in Vero E6 cells. The mapped RNA sequence has been submitted to Genbank previously (Accession No. MW041156).

### 2.2 Vero E6 cell culture and plate preparation

Vero E6 cells were maintained in complete EMEM [EMEM supplemented with 10% heat-inactivated fetal bovine serum (Gibco; 10500-064) and 1% penicillin/streptomycin (Gibco; 15140-122)] in T175 flasks (Thermo Fisher Scientific, Waltham, MA, USA) at 37°C with 5% CO_2_. Cells were seeded in resting EMEM [EMEM supplemented with 10% heat-inactivated fetal bovine serum] at 1 × 10^5^ cells/well in 96-well plates (Grenier Bio-one; 655090). Plates were incubated for 20 h at 37°C with 5% CO_2_ to allow the cells to reach 100% confluence. The resting minimal medium was removed, and the cells were used for downstream applications.

### 2.3 Concentration–response for remdesivir and ivermectin against SARS-CoV-2

Vero E6 cells were treated in triplicate with either drug in minimal medium at 25.00 µM, 8.33 µM, 2.78 µM, 0.93 µM, 0.31 µM, 0.10 µM and 0.03 µM (DMSO maintained at 0.25%) or control media, as appropriate. The plates were incubated at 37°C with 5% CO_2_ for 2 h. The minimal media containing the experimental compounds or the control media was then removed. Fifty microlitres of minimal media containing SARS-CoV-2 (MOI 0.05), 100 µL of 2× semi-solid media and 50 µL of minimal media containing experimental compounds and control media was added to each well, as appropriate. After 48 h, 4% v/v paraformaldehyde was added to each well and the plate was incubated for 1 h at room temperature. The medium was removed and cells were stained with crystal violet. Cells were washed three times with water, and cytopathic viral activity was determined by measuring the absorbance of each well at 590 nm using a Varioskan LUX microplate reader (Thermo Fisher Scientific).

Automated data quality control and data analyses were performed. For quality control, for the viral control, any well which had a log-transformed value that was 2 standard deviations above the mean of all log-transformed viral controls was excluded. Similarly, for the non-viral control, any well which had a log-transformed value that was 2 standard deviations below the mean of all log-transformed non-viral controls was excluded. If two or more wells were excluded on this basis for either control, the plate was voided and no further analysis was performed. Next, Z′ was calculated for each plate using the uninfected/untreated controls and infected/untreated controls according to Equation 1:

(1) $$Z^{\prime }=1-\frac{3\left({\hat{\sigma }}_{n}+{\hat{\sigma }}_{v}\right)}{\left|{\hat{\mu }}_{n}-{\hat{\mu }}_{v}\right|}$$

where ${\hat{\sigma }}_{n}$ and ${\hat{\sigma }}_{v}$ represent the standard deviation of the non-viral and viral controls respectively, while ${\hat{\mu }}_{\;n}$ and ${\hat{\mu }}_{\;v}$ represent the corresponding means of these controls. Drug activity was expressed as a percentage of inhibition of viral growth relative to the uninfected/untreated control (100% inhibition of viral cytopathic activity) and the infected/untreated control (0% inhibition of viral cytopathic activity) on that plate. Half maximal effective concentration (EC_50_) and 90% maximal effective concentration (EC_90_) were calculated for each compound that generated a robust, converged four-parameter fit according to Equation 2:

(2) $$E=\frac{E_{M\text{ax}}\cdot C^{h}}{E_{50}^{h}\cdot C^{h}}$$

where E is the drug effect at any given concentration (*C*), E_max_ is the maximal level of viral inhibition (0–100%), EC_50_ is the concentration required to achieve half of this maximal inhibition, and h represents the hill slope which describes the steepness of the concentration–effect relationship.

Compounds that did not achieve ≥50% viral inhibition were deemed inactive without fitting. Concentrations that were deemed toxic, as evidenced by >20% (approximately two standard deviations of all data) drop in absorbance with concentration increase coupled with evidenced toxicity in drug controls, were excluded from fitting analysis.

### 2.4 FICI for remdesivir–ivermectin combinations against SARS-CoV-2

Following assessment of the inhibitory effect (EC_50_) of remdesivir and ivermectin monotherapy on the cytopathic viral activity of SARS-CoV-2, FICI was determined using the isobologram method developed by Berenbaum [16] using data from three independent biological replicates. Drug stocks were created in DMSO to provide a stock sufficient to produce a top concentration of 25 µM for each biological replicate. Drugs were combined to generate mixed ratios of 1:0, 0.8:0.2, 0.6:0.4, 0.4:0.6, 0.2:0.8 and 0:1.0. Fixed ratios were then diluted across a concentration range 1:2 (DMSO maintained at 1%) to generate concentration–response data for each ratio, as described previously. Ratio dilutions were performed in a single 2-mL deep-well plate, and added in parallel to three 96-well plates for each biological replicate. One additional plate which was not inoculated with virus was included to observe drug toxicity. Compound incubation and viral addition was performed as described above. *Z*′ was calculated and quality control was implemented as above. Interpretation of FICI (≤0.5 = synergistic; >4.0 = antagonistic; >0.5–4 = no interaction) was based on guidance provided by the *Journal of Antimicrobial Chemotherapy* [17].

### 2.5 Checkerboard combinations for remdesivir–ivermectin combinations against SARS-CoV-2

For robustness, a second method to assess pharmacodynamic drug combination interaction was utilized. Drug stocks were created by serial dilution. Compounds and controls were mixed 1:1 (DMSO maintained at 1%) to generate data for each combination alone and in combination. Remdesivir was studied at 10 µM, 5 µM, 2.5 µM, 1.25 µM and 0.63 µM, and ivermectin was studied at 5 µM, 2.5 µM, 1.25 µM, 0.63 µM and 0.31 µM. These concentrations were selected as they were determined not to cause cell toxicity to Vero E6 cells. Ratio dilutions were performed in a single 2-mL deep-well plate, and added in parallel to three 96-well plates for each biological replicate. Compound incubation and viral addition was performed as described above. *Z*′ was calculated and quality control was implemented as above. Data were analysed using SynergyFinder and a summary synergy score was generated (>10 synergistic, −10 to +10 additive, and <−10 antagonistic) [18].

## 3 Results

**Fig. 1:**
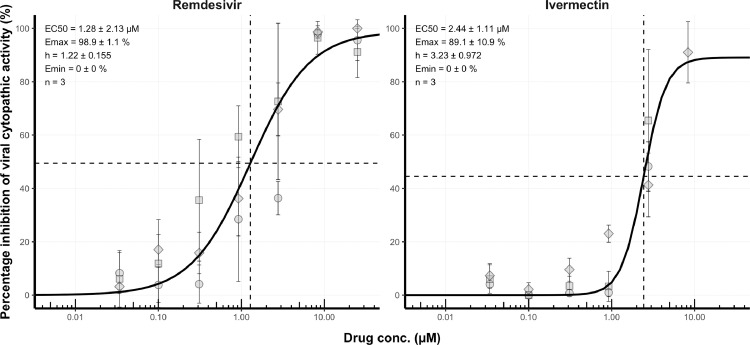
Concentration–effect relationship for the inhibition (%) of severe acute respiratory syndrome coronavirus-2 cytopathic activity for remdesivir and ivermectin. For each compound, activity was expressed relative to uninfected/untreated controls (100% inhibition of viral cytopathic activity) and infected/untreated controls (0% inhibition of viral activity). For each compound, activity was assessed at 25.00 µM, 8.33 µM, 2.78 µM, 0.93 µM, 0.31 µM, 0.10 µM and 0.03 µM in triplicate. Data points impacted by drug toxicity were removed automatically. Non-linear regression using an E_max_ model was performed on data taken from three independent biological replicates in order to generate concentration–effect predictions (solid black lines). For each compound, half maximal effective concentration **(**EC_50_) values, hillslope and replicate number (*n*) are shown. Dashed lines represent EC_50_ of each compound. Squares, diamonds and circles represent individual biological replicates, and error bars represent standard deviation calculated from technical triplicates.

This study assessed the capacity of remdesivir and ivermectin combinations to inhibit the *in vitro* cytopathic activity of SARS-CoV-2. First, the activity of each compound in isolation was determined. For plates included in concentration–response analyses, the median signal to noise ratio was 29.3 and the median Z′ was 0.43 for concentration–response plates (Table 1). For each compound, a robust four-parameter fit was generated (Figure 1). EC_50_ was 2.4 ± 1.1 µM for ivermectin and 1.3 ± 2.1 µM for remdesivir (geometric mean ± geometric standard deviation).

**Table 1:** Assay performance measures.

	Concentration–response	Isobologram	Checkerboard
Total number of plates analysed	6	9	9
Signal to noise ratio (median [range])	29.3 (19.6–39.4)	26.4 (13–37.3)	23.6 (9.2–68.5)
Signal to background ratio (median [range])	2.6 (1.9–4.1)	1.9 (1.6–2.2)	2.7 (2.3–3.5)
Z′ (median [range])	0.43 (0.39–0.76)	0.49 (0.18–0.7)	0.62 (0.2–0.9)

**Fig. 2:**
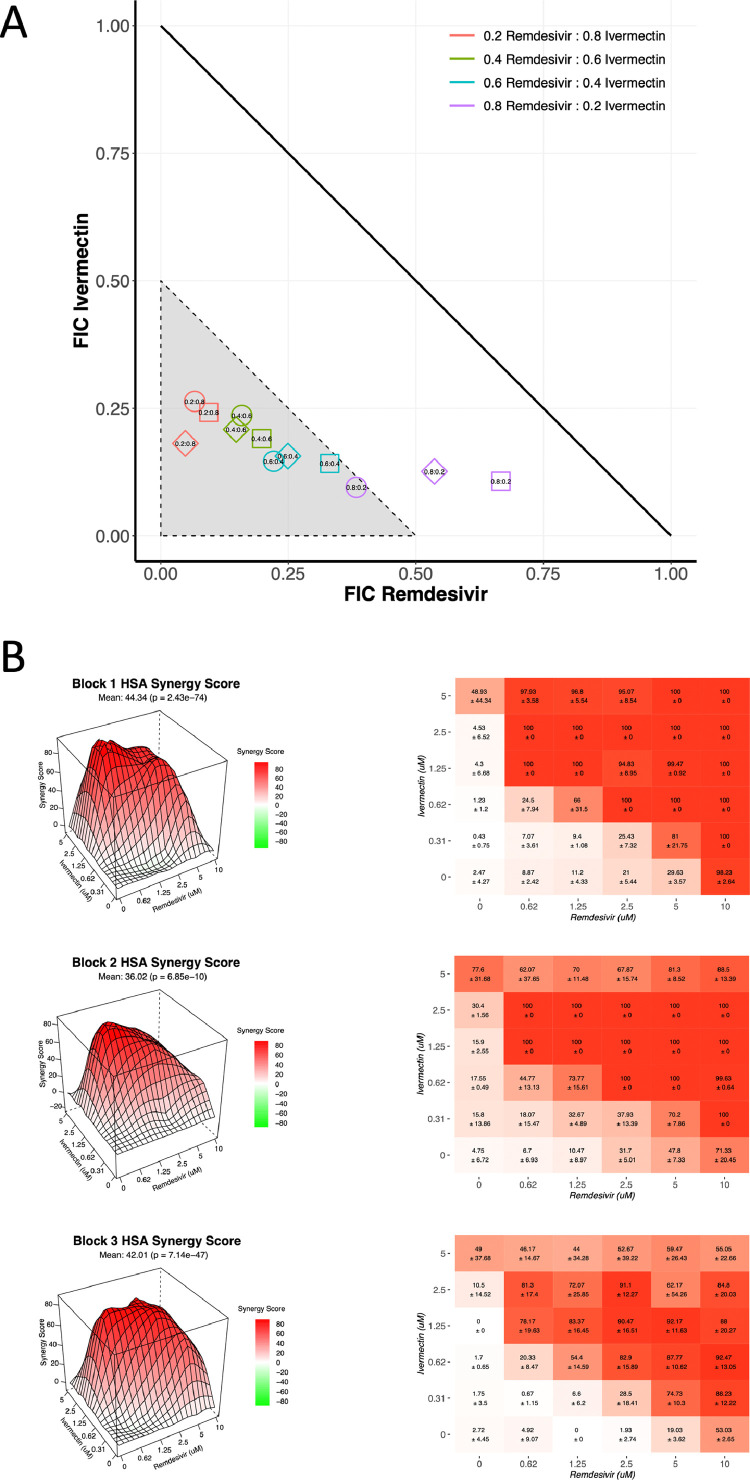
Ivermectin and remdesivir display synergistic interaction. (A) Using half maximal effective concentration **(**EC_50_) values, ranges of ivermectin and remdesivir were analysed for synergy. Data are presented for fixed concentrations at 25 µM (corresponding to 1.0), 20 µM (0.8), 15 µM (0.6), 10 µM (0.4) and 5 µM (0.2). The area indicating synergy [fractional inhibitory concentration (FIC) ≤0.5] is shown in grey. Squares, diamonds and circles represent individual biological replicates, each derived from technical triplicates. (B) Three-dimensional (3D) visualization of compound integration based on the highest single agent (HSA) synergy score (left) alongside heatmap showing compound combination dose–response matrices (right). 3D visualizations and matrices are shown for individual biological replicates, each derived from technical triplicates.

Next, the combination interaction between remdesivir and ivermectin was determined by isobologram. The median signal to noise ratio was 26.4 and the median Z′ was 0.49 for isobologram plates (Table 1). The 0.2:0.8 [remdesivir:ivermectin (5 µM:20 µM)] ratio, 0.4:0.6 ratio (10 µM:15 µM) and 0.6:0.4 (15 µM:10 µM) ratio demonstrated synergy (FICI<0.5) across all three biological replicates (Figure 2). For the 0.8:0.2 (20 µM:5 µM) ratio, just one biological replicate met the defined threshold of synergy (Figure 2). The other two biological replicates did, however, exceed the predicted effect assuming a purely additive relationship (Figure 2A).

The synergistic interaction was confirmed using interaction potency models using the SynergyFinder platform [18]. The median signal to noise ratio was 23.6 and the median Z′ was 0.62 for checkerboard plates (Table 1). All four integrated synergy models determined that interactions between remdesivir and ivermectin were synergistic with synergy scores that far exceeded the threshold for synergy (Table 2 and Figure 2B).

**Table 2:** SynergyFinder synergy score summary table for remdesivir and ivermectin.

	Mean synergy score (median [range])
ZIP	35.33 (28.01–40.84)
HSA	40.25 (36.02–44.34)
Leowe	26.34 (26.04–30.45)
Bliss	37.77 (27.61–41.69)

ZIP, zero interaction potency; HSA, highest single agent.

## 4 Discussion

This study found a synergistic interaction between remdesivir and ivermectin, both approved by the US Food and Drug Administration, resulting in enhanced *in vitro* antiviral activity against SARS-CoV-2. Although combination therapy offers a number of advantages compared with monotherapy, genuine descriptions of synergy are relatively infrequent [19]. Despite thousands of combination experiments having been performed, there have been very few reports of validated synergistic interactions against SARS-CoV-2 [4,20].

At this stage, the mechanism underpinning the synergistic interaction between remdesivir and ivermectin is unclear; however, both drugs have previously been shown to inhibit SARS-CoV-2 replication [7,12]. Given that remdesivir is known to inhibit the RNA-dependent, RNA polymerase [21], it will be of interest to investigate whether ivermectin confers synergy by inhibiting an undefined alternative but complimentary role in RNA synthesis. Ivermectin has been shown to inhibit replication of HIV-1 and dengue through inhibition of importin-β-mediated nuclear transport [22]. *In silico* predictions suggest that ivermectin may interact with host-cell proteins such as importins, which are required for nuclear transport, as well as viral proteins, including Nsp13 helicase and M^pro^ protease, which facilitate replication and translation of SARS-CoV-2 [23]. Further mechanistic studies will be required to determine the validity of *in silico* predictions.

Special care was taken to assess *in vitro* activity across concentrations that likely cover the physiological exposure of remdesivir and ivermectin in human plasma and lung tissue. In humans, a single 225-mg dose of remdesivir has been shown to produce a plasma C_max_ of approximately 4000 ng/mL [24], exceeding its *in vitro* EC_50_ (1.3 ± 2.1 µM). In humans, a high dose of 600 µg/kg/day of ivermectin has been shown to produce a plasma C_max_ of 120 ng/mL [25], which is much less than its *in vitro* EC_50_ (2.4 ± 1.1 µM). The C_max_ of remdesivir in lung epithelial lining fluid (ELF) has not been established, and it is likely that these concentrations are important in terms of clinical activity. Poor exposure in lung ELF may well explain the limited impact of remdesivir in the clinic [8]. Interestingly, concentrations of ivermectin are predicted to be some three-fold higher in the lung than in plasma [26]; however, even at these levels, ivermectin fails to meet its *in vitro* EC_50_ and no data are presented here, or elsewhere, that would support the clinical application of ivermectin for the treatment of SARS-CoV-2 infection. Given that 88–93.6% of remdesivir [27] and 93.2% of ivermectin [28] is protein-bound, the availability of unbound drug at target sites is predicted to be considerably less than the reported values based on total drug concentrations.

Data presented here demonstrate that remdesivir administered in combination with ivermectin enhances *in vitro* antiviral activity. As described above, with respect to ivermectin, due to insufficient exposure of unbound drug at the target site, this combination strategy does not represent a clinically tractable therapeutic strategy. In addition, the differing routes of administration would likely impact the ability to achieve therapeutic concentrations of both drugs simultaneously. Further investigations are now required to determine whether the observed synergistic interaction can be replicated in animal disease models and with drugs that share similar modes of action, such as, for example, the orally bioavailable polymerase inhibitors, favipiravir or molnupiravir. The underpinning mechanisms for this synergy warrant further investigation so that this pharmacodynamic phenomenon can be exploited for the development of optimal drug combinations.

## Appendix Supplementary materials

Image, application 1
