# Supplementary material for: Remdesivir–ivermectin combination displays synergistic interaction with improved in vitro activity against SARS-CoV-2
Source: Int J Antimicrob Agents. 2022 Mar;59(3):106542. doi: 10.1016/j.ijantimicag.2022.106542 (PMC8801767; doi:10.1016/j.ijantimicag.2022.106542)
Supplement: Supplementary file 1 [file mmc1.docx]

**

***Supplementary Figure 1. Individual Z’ values for all plates included in analysis.*** Z′ was calculated for each plate using the uninfected/untreated controls and infected/untreated as defined in ‘Equation 1’. Data are shown for all concentration-response (C-R), isobologram (I) and checkerboard (C) plates included in analysis.

***
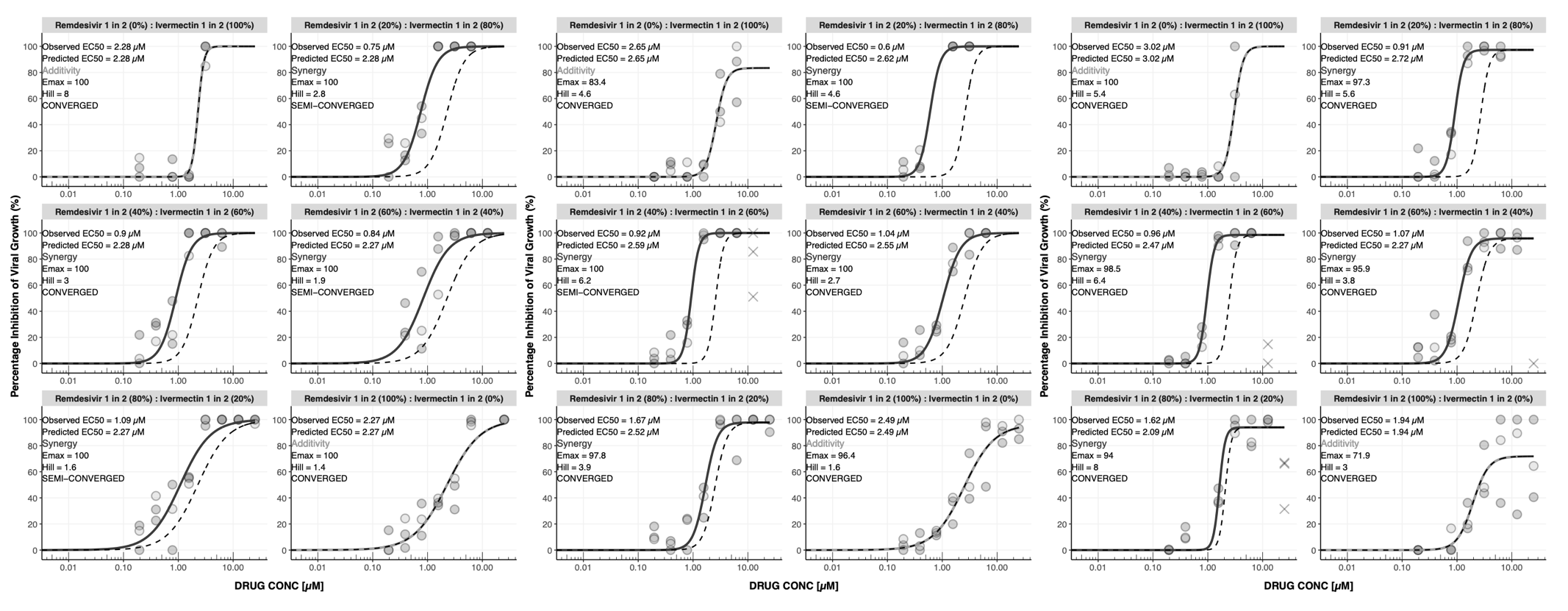
***

***Supplementary Figure 2. Ivermectin and remdesivir assessment of synergistic interaction.*** *Using EC_50_ values ranges of ivermectin and remdesivir were analysed for synergy from 7-point sigmoidal curves. Data are presented for fixed concentrations at 25 µM (corresponding to 1.0), 20 µM (0.8), 15 µM (0.6), 10 µM (0.4) and 5 µM (0.2). Each plot represents data collected from a single biological replicate and each point represents a single technical replicate.*
